# Dieckol Isolated from *Eisenia bicyclis* Ameliorates Wrinkling and Improves Skin Hydration via MAPK/AP-1 and TGF-β/Smad Signaling Pathways in UVB-Irradiated Hairless Mice

**DOI:** 10.3390/md20120779

**Published:** 2022-12-14

**Authors:** Jae-Min Kim, Kyung-Sook Chung, Young-Seo Yoon, Seo-Yun Jang, So-Won Heo, Geonha Park, Young-Pyo Jang, Hye-Shin Ahn, Yu-Kyong Shin, Sun-Hee Lee, Kyung-Tae Lee

**Affiliations:** 1 Department of Pharmaceutical Biochemistry, College of Pharmacy, Kyung Hee University, 26 Kyungheedae-ro, Seoul 02447, Republic of Korea; 2 Department of Biomedical and Pharmaceutical Science, Graduate School, Kyung Hee University, 26 Kyungheedae-ro, Seoul 02447, Republic of Korea; 3 Department of Fundamental Pharmaceutical Science, Graduate School, Kyung Hee University, 26 Kyungheedae-ro, Seoul 02447, Republic of Korea; 4 Department of Life and Nanopharmaceutical Science, Graduate School, Kyung Hee University, 26 Kyungheedae-ro, Seoul 02447, Republic of Korea; 5 Department of Integrated Drug Development and Natural Products, Graduate School, Kyung Hee University, 26 Kyungheedae-ro, Seoul 02447, Republic of Korea; 6 Department of New Material Development, COSMAXBIO, Seongnam 13486, Republic of Korea

**Keywords:** dieckol, photoaging, ultraviolet B, MMPs, collagen, MAPK, TGF-β, Smad, hyaluronic acid

## Abstract

Repetitive exposure to ultraviolet B (UVB) is one of the main causes of skin photoaging. We previously reported that dieckol isolated from *Eisenia bicyclis* extract has potential anti-photoaging effects in UVB-irradiated Hs68 cells. Here, we aimed to evaluate the anti-photoaging activity of dieckol in a UVB-irradiated hairless mouse model. In this study, hairless mice were exposed to UVB for eight weeks. At the same time, dieckol at two doses (5 or 10 mg/kg) was administered orally three times a week. We found that dieckol suppressed UVB-induced collagen degradation and matrix metalloproteinases (MMPs)-1, -3, and -9 expression by regulating transforming growth factor beta (TGF-β)/Smad2/3 and mitogen-activated protein kinases (MAPKs)/activator protein-1 (AP-1) signaling. In addition, dieckol rescued the production of hyaluronic acid (HA) and effectively restored the mRNA expression of hyaluronan synthase (HAS)-1/-2 and hyaluronidase (HYAL)-1/-2 in UVB-irradiated hairless mice. We observed a significant reduction in transepidermal water loss (TEWL), epidermal/dermal thickness, and wrinkle formation in hairless mice administered dieckol. Based on these results, we suggest that dieckol, due to its anti-photoaging role, may be used as a nutricosmetic ingredient for improving skin health.

## 1. Introduction

The skin is the first line of protection for the body from the outer environmental challenges, such as external irritation, invasion of pathogens, and ultraviolet (UV) radiation. The skin is a complex organ with a multilayer structure composed of the epidermis, dermis, and subcutaneous fat. The epidermis contains various cells, such as melanocytes, Langerhans cells, and Merkel cells, but keratinocytes constitute 90% of epidermal skin cells [1]. The main function of keratinocytes is the skin barrier function by forming an extracellular matrix (ECM). The dermis, the skin layer between the epidermis and subcutaneous fat, consists of fibroblasts and matrix components, such as collagen, elastin, hyaluronic acid (HA), and glycoproteins [2]. Dermal collagen is one of the main structural proteins that confer strength and elasticity to the skin [3]. HA is an essential skin moisture component for maintaining healthy skin because it holds water nearly 1000 times its weight [4]. Although UVB is known to regulate hyaluronan synthase (HAS) and hyaluronidase (HYAL) for the production of HA, a key factor that mediates skin moisture [5,6,7], the effect of UVB radiation on HA and the exact underlying mechanisms are not well known.

Cumulative and repetitive UV exposure of the skin is directly related to photoaging by contributing to the factor responsible for cellular DNA damage, sunburns, and photocarcinogenesis [8]. Although UVB reaches the earth 10-fold less than UVA, it has the most potential for damaging the skin [9]. Excessive UVB exposure changes the structure of the skin by reducing its integrity and strength, thereby increasing water loss and wrinkle formation, and resulting in dryness and sagging [10,11]. UVB irradiation activates the generation of reactive oxygen species (ROS) in the skin and subcutaneous tissues [12]. ROS mediate the activation of receptor tyrosine kinases (RTKs) by binding to the catalytic site of receptor protein tyrosine phosphatases (RPTPs) [13]. These RPTPs are inactivated by ROS, thereby maintaining RTKs at high levels of activation [14]. Mitogen-activated protein kinases (MAPKs), the downstream signaling, phosphorylated by activated RTKs eventually activate activator protein-1 (AP-1) transcription factors which promote matrix metalloproteinase (MMP) family expression and inhibit the transcription of procollagen [3]. Overexpression of MMPs cleaves collagen fibers in the extracellular matrix and impairs the mechanical and structural functions of the skin [15]. UVB irradiation also regulates transforming growth factor beta (TGF-β) signaling, phosphorylates Smad2/3, and affects procollagen synthesis and MMP-1 transcription [16].

In the marine environment, brown seaweeds contain abundant bioactive compounds, such as polysaccharides, polyphenols, phytosterols, and carotenoids [17]. By identifying the beneficial effects of native compounds from *Eisenia bicyclis*, a type of brown algae, numerous studies have reported its antioxidant [18,19], antibacterial [20], anti-inflammatory [21], and neuroprotective effects [22]. Similarly, our recent study showed the antioxidant and anti-photoaging effects of 30% ethanol extract from *E. bicyclis*, and we found that dieckol showed potent protective effects on the production of MMP-1 and procollagen in UVB-irradiated Hs68 human fibroblasts [23]. Therefore, we hypothesized that dieckol has anti-photoaging effects and investigated the mechanism of wrinkle formation and dehydration in UVB-irradiated hairless mice treated with dieckol.

## 2. Results

### 2.1. Dieckol Suppresses Skin Wrinkle Formation on the Dorsal Skin of UVB-Irradiated HR-1 Mice

**Figure 1:**
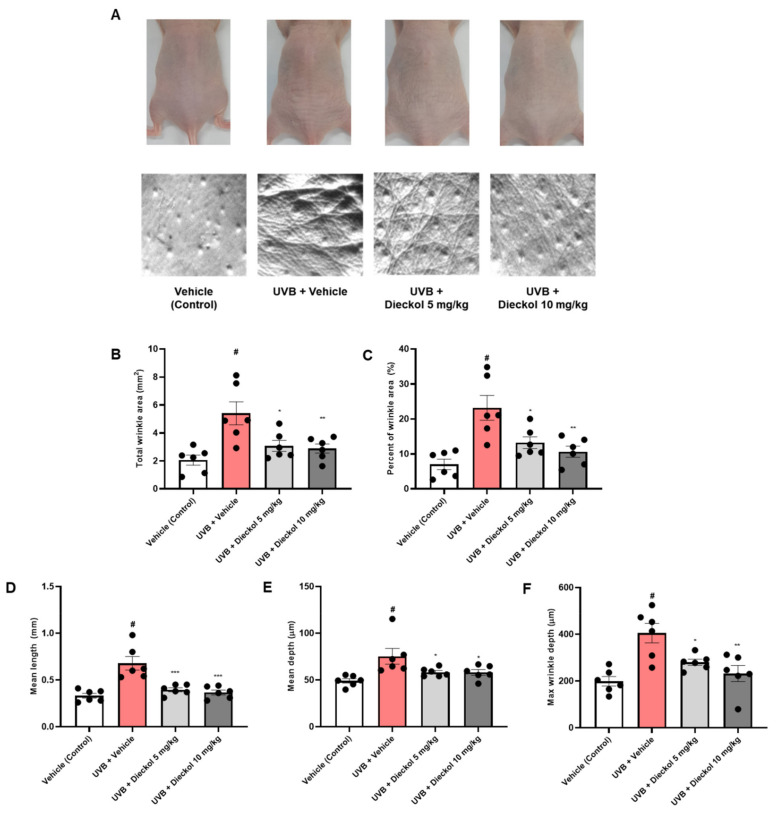
Effects of dieckol on the prevention of wrinkle formation in the UVB-irradiated HR-1 mice. HR-1 mice were administered dieckol (5 or 10 mg/kg) three times per week for 8 weeks after UVB irradiation (60 mJ/cm^2^ to 120 mJ/cm^2^). (**A**) Representative images of dorsal skin of mice and replica skin. The wrinkles on replica skin were measured by Visioline^®^ VL650; (**B**) total wrinkle area; (**C**) percent of wrinkle area; (**D**) mean length; (**E**) mean depth; (**F**) max wrinkle depth. Data are expressed as the mean ± SEM (*n* = 6). *^#^ p* < 0.05 vs. the vehicle-treated control group; * *p* < 0.05, ** *p* < 0.01, and *** *p* < 0.001 vs. UVB only-treated group.

To assess the protective effect of dieckol on UVB-irradiated HR-1 mice, HR-1 mice were gradually exposed to UVB, and dieckol (5 or 10 mg/kg) was administered orally for 8 weeks. As shown in the replica images in Figure 1A, the number and depth of wrinkles in HR-1 mice exposed to UVB increased significantly compared to non-irradiated HR-1 mice. However, dieckol treatment markedly alleviated wrinkle formation. In addition, UVB irradiation markedly increased the total wrinkle area, percentage of wrinkle area, mean length and depth, and maximum wrinkle depth, whereas oral administration of dieckol (5 or 10 mg/kg) significantly (*p* < 0.05) reduced the wrinkle parameters (Figure 1B–F).

### 2.2. Dieckol Protects against Skin Thickening and Water Loss on the Dorsal Skin of UVB-Irradiated HR-1 Mice

**Figure 2:**
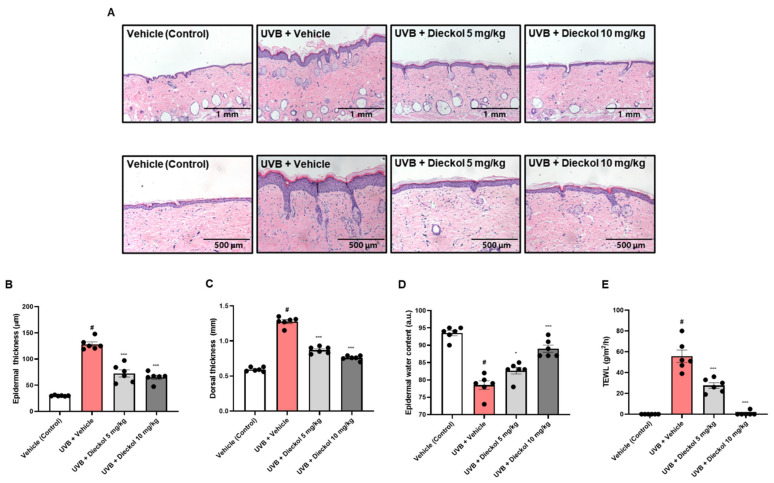
Effects of dieckol on skin thickening and loss of water content in UVB-irradiated HR-1 mice. HR-1 mice were administered dieckol (5 or 10 mg/kg) three times per week for 8 weeks after UVB-irradiation (60 mJ/cm^2^ to 120 mJ/cm^2^). (**A**) Hematoxylin eosin-stained sections of the dorsal skin. Scale Bars = 1 mm (upper); 500 μm (lower); (**B**) epidermal thickness was calculated by averaging the thickness measured at three locations in each section; (**C**) dorsal skin thickness was measured with a digital caliper; (**D**,**E**) the epidermal water content and TEWL were measured with GPSkin Barrier^®^. Data are expressed as the mean ± SEM (*n* = 6). ^#^ *p* < 0.05 vs. the vehicle-treated control group; * *p* < 0.05 and *** *p* < 0.001 vs. UVB only-treated group.

Since repetitive UVB exposure leads to increased epidermal thickness and polymorphism of keratinocytes in skin tissues [24], we analyzed whether dieckol affected the UVB irradiation-induced skin thickness by H&E staining of dorsal skin in HR-1 mice. H&E staining revealed a thickening of the dorsal skin in the UVB-only-treated group compared to the vehicle-treated control group; however, oral administration of dieckol decreased dorsal skin thickening (Figure 2A). The thickness of the dorsal epidermis was measured at random locations. The average epidermal thickness of the UVB-only-treated group (128.09 ± 4.33 μm) markedly increased from that of the control group (29.84 ± 0.38 μm), which was significantly (*p* < 0.001) reduced to 72.48 ± 6.89 μm, and 65.17 ± 4.02 μm in the dieckol-treated group at 5 or 10 mg/kg, respectively (Figure 2B). As shown in Figure 2C, the oral administration of dieckol also significantly (*p* < 0.001) suppressed dorsal skin thickness (0.87 ± 0.02 and 0.76 ± 0.01 mm at 5 and 10 mg/kg, respectively) that had increased due to UVB exposure (1.28 ± 0.01 mm). To investigate the effects of dieckol on skin moisturizing factors, we measured epidermal hydration and transepidermal water loss (TEWL) before euthanasia. UVB irradiation significantly reduced epidermal water contents compared to the control group (93.5 ± 0.76 vs. 78.5 ± 12.3, *p* < 0.05), whereas dieckol significantly (*p* < 0.05) recovered the value (82.67 ± 0.99 and 89 ± 1.03 at 5 and 10 mg/kg, respectively, Figure 2D). Moreover, oral administration of dieckol completely restored UVB irradiation-induced TEWL (55.67 ± 5.96 g/m^2^/h) to the vehicle-treated group at 10 mg/kg administration of dieckol (0.83 ± 0.83 g/m^2^/h, Figure 2E).

### 2.3. Dieckol Inhibits Collagen Degradation in the Dorsal Skin of UVB-Irradiated HR-1 Mice

**Figure 3:**
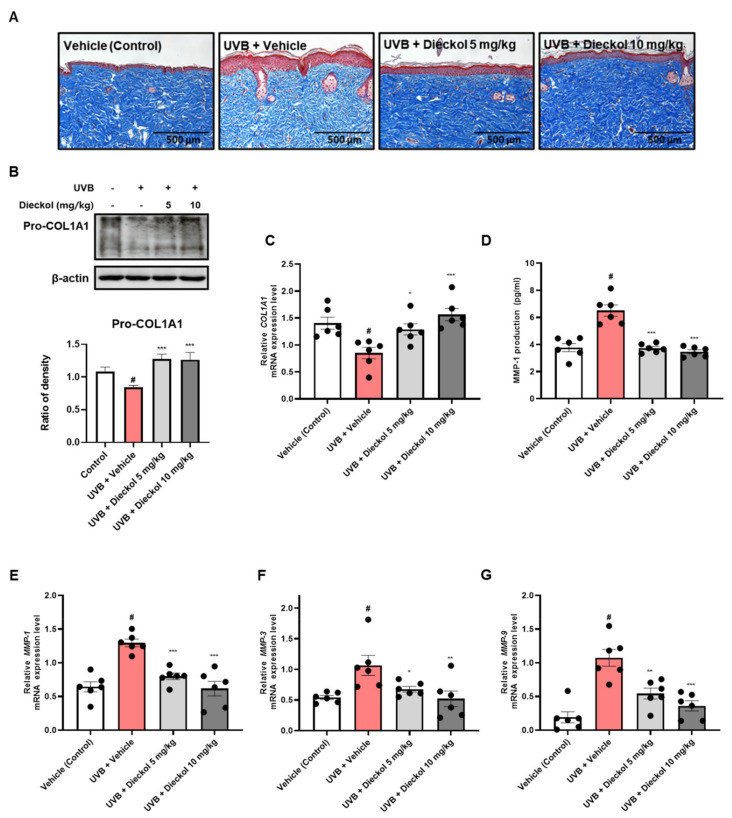
Protective effects of dieckol against UVB-induced dehydration in HR-1 mice. HR-1 mice were administered dieckol (5 or 10 mg/kg) three times per week for 8 weeks after UVB-irradiation (60 mJ/cm^2^ to 120 mJ/cm^2^). (**A**) Masson’s trichrome-stained sections of the dorsal skin. Scale Bars = 500 μm; (**B**) the protein expression level of pro-COL1A1 was evaluated by Western blot analysis. β-actin is used as an internal control; (**C**) the mRNA expression level of *COL1A1* was quantified through qRT-PCR; (**D**) the contents of MMP-1 in dorsal skin tissue were measured by the MMP-1 ELISA kit. (**E**–**G**) The mRNA expression levels of *MMP-1, -3,* and *-9* were analyzed by qRT-PCR. Data are expressed as the mean ± SEM (*n* = 6). ^#^ *p* < 0.05 vs. the vehicle-treated control group; * *p* < 0.05, ** *p* < 0.01 and *** *p* < 0.001 vs. UVB only-treated group.

With skin aging, loss of collagen leads to wrinkling formation in skin appearance [25]. We determined the effect of dieckol on UVB irradiation-induced collagen degradation using Masson’s trichrome staining in HR-1 mice. The UVB only-treated group showed a reduction in and broken collagen fibers. However, dieckol treatment significantly diminished UVB-induced collagen degradation and the structural density of collagen fibers in the skin (Figure 3A). As type I collagen is the most abundant subtype of collagen found within the connective tissue of the skin [26], we further investigated whether dieckol inhibited the degradation of the collagen type I α 1 chain (COL1A1) using Western blotting and qRT-PCR. As shown in Figure 3B,C, UVB irradiation-reduced expression levels of pro-COL1A1 protein and COL1A1 mRNA were improved by dieckol administration. The zinc-containing endopeptidases MMP-1, -3, and-9 are secreted by dermal fibroblasts in response to UV radiation [27]. As MMPs can degrade fibrillar collagen in the connective tissue of the skin, we evaluated the effect of dieckol on the production of MMP-1 and mRNA expression levels of MMP-1, -3, and -9 in UVB-irradiated HR-1 mice. As shown in Figure 3D, MMP-1 production was more significantly increased in the UVB-only-treated group (6.50 ± 0.42 pg/mL, *p* < 0.05) than in the control group (3.77 ± 0.30 pg/mL), whereas it was effectively decreased in the dieckol-treated group (3.72 ± 0.13 and 3.46 ± 0.15 pg/mL at 5 and 10 mg/kg respectively, *p* < 0.001). Similar to the results of MMP-1 production, the mRNA expression levels of MMP-1, -3, and -9 were upregulated in the UVB-only-treated group; however, dieckol administration effectively inhibited these elevations. (Figure 3E–G). These results suggest that dieckol exerts moisturizing effects on skin exposed to UVB by preventing collagen degradation and inhibiting the production of MMP and expression mRNA in UVB-irradiated HR-1 mice.

### 2.4. Dieckol Prevents UVB-Induced Activation of the MAPK/AP-1 and TGF-β/Smad2/3 Signaling Pathways in the Dorsal Skin of UVB-Irradiated HR-1 Mice

**Figure 4:**
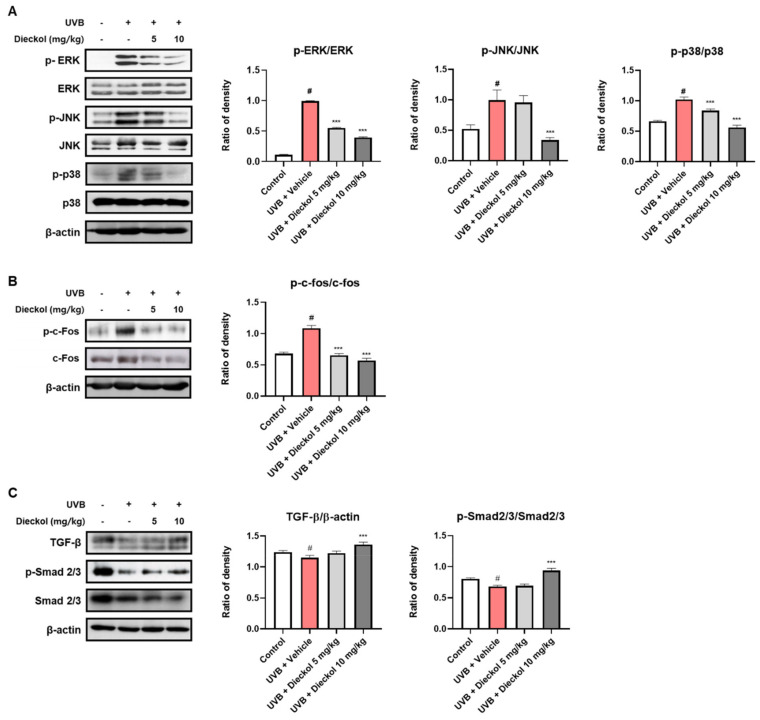
Effects of dieckol on the MAPK and TGF-β/Smad 2/3 signaling pathway in UVB-irradiated HR-1 mice. HR-1 mice were administered dieckol (5 or 10 mg/kg) three times per week for 8 weeks after UVB irradiation (60 mJ/cm^2^ to 120 mJ/cm^2^). The protein expression and phosphorylation level of (**A**) MAPKs (ERK, JNK, and p-38); (**B**) p-c-Fos, c-Fos, and (**C**) TGF- β/Smad 2/3 were evaluated by Western blot analysis and relative densities. β-actin is used as an internal control. Data are expressed as the mean ± SEM (*n* = 6). ^#^ *p* < 0.05 vs. the vehicle-treated control group; *** *p* < 0.001 vs. UVB only-treated group.

MAPK signaling contributes to the homeostasis of the skin barrier by inhibiting TGF-β signaling, a major regulator for the production of procollagen type I [28]. Our results showed that UVB irradiation promoted MAPK phosphorylation, whereas dieckol effectively decreased UVB irradiation-triggered MAPK activation, particularly phospho-level of JNK and p-38, in the dorsal skin of HR-1 mice (Figure 4A). Furthermore, dieckol restored the UVB-induced upregulation of both the phosphorylation and total protein expression levels of c-Fos to the control level (Figure 4B). Dieckol significantly (*p* < 0.001) restored the protein expression of TGF-β and the phosphorylation levels of Smad 2/3 in the 10 mg/kg-treated group (Figure 4C). Based on these results, we suggest that dieckol attenuates MMP expression and improves procollagen synthesis by regulating MAPKs, p-c-Fos, c-Fos, TGF-β, and Smad 2/3 signaling in UVB-irradiated hairless mouse skin.

### 2.5. Dieckol Restores Hyaluronic Acid Production and HAS-1/-2 and HYAL-1/-2 mRNA Expression in the Dorsal Skin of UVB-Irradiated HR-1 Mice

**Figure 5:**
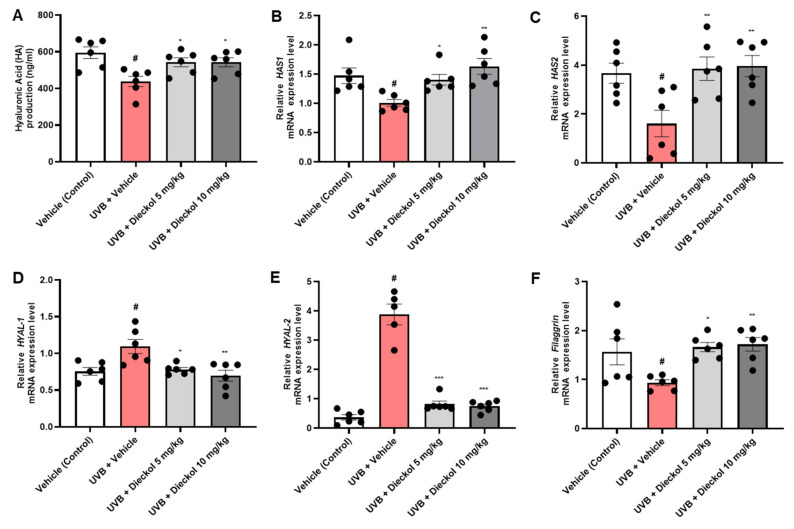
Effects of dieckol on hyaluronic acid content, hyaluronic acid synthase, and hyaluronidase mRNA levels in UVB-irradiated HR-1 mice. HR-1 mice were administered dieckol (5 or 10 mg/kg) three times per week for 8 weeks after UVB irradiation (60 mJ/cm^2^ to 120 mJ/cm^2^). (**A**) Measurement of HA production level in dorsal skin tissue; (**B**–**F**) mRNA levels of *HAS-1/-2*, *HYAL-1/-2*, and *filaggrin* were evaluated by qRT-PCR. Data are expressed as the mean ± SEM (*n* = 6). ^#^ *p* < 0.05 vs. the vehicle-treated control group; * *p* < 0.05, ** *p* < 0.01 and *** *p* < 0.001 vs. UVB only-treated group.

Since HA is a linear polysaccharide [29], it can combine with a considerable amount of moisture by regulating the skin barrier function, resulting in the maintenance of moisture homeostasis in the tissue [30]. Therefore, we measured HA production to determine the effect of dieckol on moisture protection in the dorsal skin of UVB-irradiated HR-1 mice. As shown in Figure 5A, the HA production level, which was 594.92 ± 31.77 ng/mL in the control group, was markedly reduced to 437.68 ± 28.63 ng/mL by UVB irradiation. However, HA production level was significantly improved with dieckol oral administration (542.68 ± 24.56 and 542.57 ± 25.35 ng/mL at 5 or 10 mg/kg, respectively, *p* < 0.05). HAS and HYAL are the enzymes responsible for cellular hyaluronic acid synthesis and degradation. Therefore, to evaluate the effects of dieckol on these enzymes, we measured the mRNA expression levels of *HAS-1*/*-2* and *HYAL-1*/*-2* using qRT-PCR analysis. The mRNA expression levels of *HAS-1* and *-2* decreased after UVB irradiation. However, dieckol administration significantly (*p* < 0.05) recovered *HAS-1* and *HAS-2* mRNA levels (Figure 5B,C). By contrast, the increase in *HYAL-1/-2* mRNA levels after UVB exposure was significantly (*p* < 0.05) decreased after dieckol administration (Figure 5D,E). Filaggrin is a structural protein fundamental for developing and maintaining the skin barrier [31]. We found that dieckol significantly (*p* < 0.05) recovered the *filaggrin* mRNA expression level that decreased after UVB irradiation (Figure 5F). These results indicate that dieckol prevents moisture loss in the skin by protecting against the reduction in hydration and skin barrier factors caused by UVB irradiation.

## 3. Discussion

*E. bicyclis*, a brown alga native to the coast of Japan and around Ullengdo Island in Korea, has been reported to possess anti-inflammatory and anti-photoaging properties [21,23]. We previously found that the 30% ethanol extract of *E. bicyclis* (EEB) could inhibit photoaging of the skin by regulating MAPK/AP-1 and ROS-mediated Nrf2 signaling pathways [23]. In addition, both eckol and dieckol, as active components of EEB, showed protective effects against UVB-induced MMP-1 production and degradation of procollagen type I in Hs68 cells. As dieckol had relatively more potent protective effects than eckol, we expect that dieckol is likely to be more effective than eckol in the animal model as an anti-photoaging agent. Interestingly, to our knowledge, there are no published reports on the anti-photoaging effect of dieckol in vivo. Therefore, in the present study, we investigated the anti-photoaging effects of dieckol in a hairless mouse model by measuring dorsal skin wrinkle formation, epidermal thickening, and skin dehydration, and its molecular mechanism in HR-1 hairless mice exposed to UVB.

As mouse skin has multiple layers consisting of the epidermis, dermis, and hypodermis, UVB irradiation was performed with repeated and gradually increasing intensity on the dorsal skin of mice to induce skin photoaging [32]. In this study, we found that the dieckol-treated group had alleviated wrinkle formation and skin roughness compared to the UVB-only-treated group in the dorsal skin that was continuously exposed to UVB. For objective wrinkle parameters measurement, we evaluated the total wrinkle area, percent of wrinkle area, mean length, mean depth, and max wrinkle depth using a replica taken from mice dorsal skin. We observed a significant recovery of these biomarkers in the dieckol-administered group. Epidermal and dermal thickening are cardinal defensive responses of human skin to UV, which weaken the function and condition of the skin barrier, resulting in a decrease in epidermal water content and an increase in TEWL [33,34]. Consistent with our previous findings, the UVB-only-treated group showed epidermal and dermal thickening and skin dehydration after UV irradiation [35], whereas dieckol significantly diminished epidermal and dermal thickness and repaired epidermal water content and TEWL. These results suggest that dieckol plays a pivotal photoprotective role.

UV irradiation causes skin aging because UV irradiation induces ROS production and pro-inflammatory cytokines [36,37,38]. MMPs, calcium-dependent zinc-containing endoproteases induced by ROS, are enzymes that degrade diverse types of collagens in the extracellular matrix (ECM) [39]. In the MMP family, MMP-1, known as fibroblast collagenase, is an enzyme that breaks down type I and III collagens and plays an important role in the photoaging process [28]. MMP-3, a stromelysin 1 protein, decomposes type IV collagen in the basal membrane and activates proMMP-1, a zymogen. MMP-9 is a gelatinase B, which hydrolyzes the product degraded by collagenase to a smaller size [40]. Since dieckol has been reported to reduce intracellular ROS levels in UVB-irradiated HDF [41], we evaluated whether dieckol inhibits MMP-1 production and expression in UVB-irradiated hairless mice. We observed that dieckol suppressed MMP-1 production and mRNA expression of *MMP-1*, *-3*, and *-9* induced by UVB exposure.

UV irradiation accelerates the degradation of collagen, a major structural protein in the skin, that causes elasticity reduction and wrinkle formation [3,42]. Furthermore, UV irradiation impaired procollagen synthesis via the TGF-β/Smad signaling pathway. When TGF-β binds to the TGF-β receptor and dimerizes, Smad2/3 combines with the cytoplasmic receptor domain, and its serine residue is phosphorylated. Phosphorylated Smad2/3 translocates to the nucleus and binds to the promoter sequence, which activates procollagen synthesis [43]. Our results showed that oral administration of dieckol protected against UVB-induced collagen degradation and inhibition of procollagen production by significantly restoring the TGF-β/Smad signaling pathway, especially in the 10 mg/kg administered group. AP-1 and NF-κB are major transcription factors that play crucial roles in the expression of TGF-β and MMPs [44,45]. Moreover, these transcription factors produce pro-inflammatory cytokines, which induce inflammatory responses in the skin [46,47]. Dieckol, isolated from various brown algae, has been reported to prevent the activation of NF-κB, ERK, JNK, and p-38 in HDF cells and human fibrosarcoma cells, respectively [41,48]. Accordingly, we assessed the protein phosphorylation levels of MAPK/AP-1 signaling markers in the dorsal skin of UVB-irradiated mice. We also observed a significant reduction in the levels of phosphorylated AP-1 and MAPK. These data indicate that dieckol functions as an anti-wrinkle agent and protects elasticity by decreasing *MMP-1*, *-3*, and *-9* expression, which breaks down fibrillary collagen via the TGF-β/Smad and MAPK signaling pathways.

HA plays crucial roles in skin metabolism, including increasing the water-binding capacity, protecting cells from radicals, and having anti-inflammatory properties [49,50]. HA is a linear polysaccharide composed of repeating disaccharides of β4-glucuronic acid-β3-*N*-acetylglucosamine that can serve as a viscoelastic structure [51]. Therefore, HA is an important biomarker for evaluating elasticity and moisture in the skin. HAS isoforms, with a channel-like structure, are composed of HAS-1, HAS-2, and HAS-3, which synthesize HA chains of different Dalton sizes at the plasma membrane [52]. In the plasma membrane, there are also HA-degrading enzymes HYAL-1 and HYAL-2, which can cleave HA into smaller units [53]. Our study showed that dieckol decreased the mRNA expression of *HYALs* and restored HA production and mRNA expression of *HASs* in the UVB-exposed skin of mice. Maintaining the structural integrity of the stratum corneum is crucial for regulating water retention in the deeper stratum of the skin. Thus, we further investigated filaggrin among various factors that function as a skin barrier. Filaggrin bound to keratin filaments provides mechanical strength and increases the water-holding capacity of the skin [54,55]. In this study, although UVB irradiation diminished the expression of *filaggrin* mRNA, treatment with dieckol effectively ameliorated *filaggrin* mRNA levels in the dieckol group. These results indicate that oral administration of dieckol prevented UVB-induced HA degradation by UVB irradiation in the skin of hairless mice by regulating HA-related *HAS-1/-2* and *HYAL-1/-2* enzymes. In addition, filaggrin, which was rescued by dieckol, suppressed water loss in the skin by strengthening the skin barrier.

In conclusion, to validate and expand the anti-photoaging study of dieckol in a UVB-induced hairless mouse model, we studied the protective effects of dieckol on the production of procollagen and HA and upstream of the TGF-β/Smad signaling pathway enzymes that contribute to the homeostasis of HA. In this study, our data demonstrated that dieckol improved wrinkles and skin hydration, even when made up of poly layers of the skin, including various kinds of skin cells. In addition, to confirm the safety of oral administration of dieckol, we measured the plasma levels of glutamic oxaloacetic transaminase (GOT), glutamic pyruvic transaminase (GPT), blood urea nitrogen (BUN), and creatinine. Biochemical assays showed that the levels in the dieckol-treated group were similar to those in the vehicle-treated control group. Accordingly, dieckol administration did not cause hepatotoxicity or nephrotoxicity (Figure S1).

## 4. Materials and Methods

### 4.1. Preparation of Dieckol from E. bicyclis

**Figure 6:**
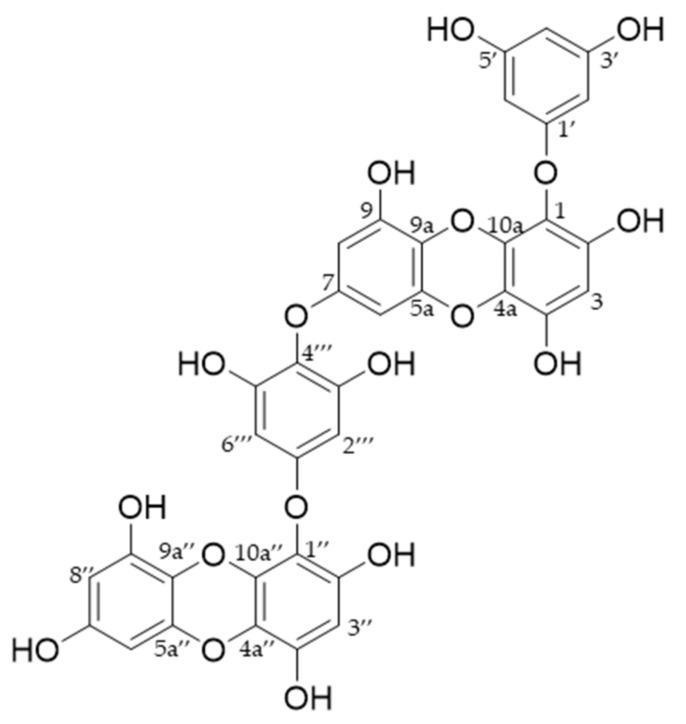
Structure of dieckol.

*E. bicyclis* was obtained as previously reported [23] and the EEB (430.0 g) was dissolved in water to set a concentration of 37.4 brix. The sample solution was coated into a Silicagel 60 (40–63 μm, Sigma Aldrich In., St Louis, MO, USA) and extracted by ethyl acetate three times. The EEB-EA was fractionated on a Diaion HP-20 (Mitsubishi chemical corporation, Tokyo, Japan) column, starting with 10% MeOH followed by 100% MeOH as an eluent, giving five fractions (Fr-1–Fr-5). Fraction 3 was subjected to a Lichroprep RP 18 (40–63 μm, Merck, Darmstadt, Germany) column eluted with 70% MeOH to afford seven subfractions (Fr-3a–Fr-3g). All fractions were compared with standard dieckol with TLC and HPLC during the isolation process to isolate a large amount of only dieckol from the EEB. Therefore, we already recognized that fraction 3d contained a lot of dieckol. Fraction 3d was subjected to a Sephadex lh-20 (10–25 μm, GE Healthcare Bio-Science AB, Helsinki, Sweden) column eluted with 20–100% MeOH to afford three subfractions (Fr-3d1–Fr-3d3). The Fr-3d2 yielded the dieckol (4.0g, Figure 6) and its purity (>97%) was determined by HPLC (Figure S2). The structure of dieckol was identified by comparing the ^1^H NMR [18], ^13^C NMR [56,57], and HPLC-ESI-TOF-MS spectral data with those in the existing literature (Figures S3–S5).

### 4.2. Animals

HR-1 hairless mice (male, 5 weeks old) were supplied from SLC Inc. (SLC Inc., Shizuoka, Japan) and housed in a climate-controlled room (at 22 ± 1 °C and 50 ± 10% humidity) under a 12 h light/12 h dark cycle and provided food and water ad libitum. All animal experiments were conducted following institution-approved Animal Care and Use guidelines of the Kyung Hee University (KHSASP-22-511).

### 4.3. Sample Treatment and UVB Irradiation

Before starting the study, the mice were acclimated for 1 week and then placed randomly into four groups (n = 6/group): vehicle-treated group (control), UVB + vehicle-treated group, and UVB + dieckol-treated groups (5 or 10 mg/kg. p.o.). Dieckol was suspended in a vehicle (Ethanol: Kolliphor^®^ EL: Sterilized water = 1:1:18) and administered orally three times per week. The UVB-treated groups were irradiated with UVB three times a week for 8 weeks as follows: 1–3 weeks, 60 mJ/cm^2^; 4–6 weeks, 120 mJ/cm^2^; and 7–8 weeks, 180 mJ/cm^2^.

### 4.4. Analysis of Skin Wrinkle Formation

On the last day of the experiment, dorsal skin replicas of HR-1 hairless mice were made using the SILFLO kit (Monaderm, Monaco) to measure skin wrinkles. The indicators of skin wrinkles involving total wrinkle area, percent of wrinkle area, mean length, mean depth, and max wrinkle depth were analyzed with a Visioline^®^ VL 650 (Courage & Khazaka Electronics GmbH, Cologne, Germany) using skin replicas.

### 4.5. Histological Analysis

Dorsal skin tissue-embedded paraffin blocks were sliced and then stained with hematoxylin and eosin (H&E) and Masson’s trichrome for the analysis of skin layer and collagen fiber changes, respectively.

### 4.6. Assessment of Dorsal Skin Thickness, Epidermal Water Content, and Transepidermal Water Loss (TEWL)

The dorsal skin thickness was determined using a digital caliper and skin water content and TEWL from the dorsal skin of HR-1 hairless mice were measured using a GPSkin Barrier^®^ (GPOWER Inc., Seoul, Republic of Korea) on the last day of the experiment.

### 4.7. Measurement of Matrix Metalloproteinase-1 (MMP-1) and Hyaluronic Acid Production

The production levels of MMP-1 and hyaluronic acid in the dorsal skin tissues were measured using ELISA kits (Abcam, Cambridge, UK; and R&D Systems, Minneapolis, MN, USA) according to the manufacturer’s instructions.

### 4.8. Western Blot Analysis

Protein extraction from dorsal skin tissues was carried out using a PRO-PREP (Intron Biotechnology, Seoul, Republic of Korea) with a protease and phosphatase inhibitor cocktail (Sigma Aldrich In., St Louis, MO, USA). The extracted protein concentration was determined by Bradford’s assay and bovine serum albumin (BSA) was used as a standard quantification. Proteins were separated with 8–10% sodium dodecyl sulfate–polyacrylamide gel electrophoresis (SDS–PAGE) and transferred to a polyvinylidene difluoride (PVDF) membrane. The membrane was reacted for 12 h at 4 °C with primary antibodies in 5% skim milk, and then reacted with secondary antibodies in 5% skim milk for 2 h at 20 to 25 °C. Bands were detected by enhanced chemiluminescence (ECL) substrate and visualization was performed using an ImageQuant^™^ LAS-4000 (FUJIFILM, Tokyo, Japan). Antibodies of pro-COL1A1 (sc-25973), ERK (sc-93), JNK (sc-7345), c-Fos (sc-253), and β-actin (sc-81178) were obtained from Santa Cruz Biotechnology Inc. (Dallas, TX, USA). p-ERK (#4377), p-JNK (#4668), p-p38 (#9215), p-38 (#9212), p-c-Fos (#5348), TGF-β (#3711), p-Smad 2/3 (#8828), and Smad 2/3 (#5678) were purchased from Cell Signaling Technology Inc. (Danvers, MA, USA).

### 4.9. Quantitative Real-Time Polymerase Chain Reaction (qRT-PCR) Analysis

Total RNA in dorsal skin tissues was extracted using Easy Blue^®^ kits (Intron Biotechnology, Seoul, Republic of Korea). The extracted mRNA was quantified using a NanoDrop^™^ 2000/2000c Spectrophotometer (Thermo Fisher Scientific, Waltham, MA, USA) and was used to synthesize cDNA with random oligonucleotide primers (Promega, Madison, WI, USA) and TOPscript^™^RTdryMIX (Enzynomics, Daejeon, Republic of Korea). The amplification of target genes with SYBR Premix Ex Taq (TaKaRa Bio Inc., Shiga, Japan) was evaluated by using QuantStudio 1 (Thermo Fisher Scientific, Waltham, MA, USA). The PCR primer sequences used in this study are listed in Table S1.

### 4.10. Statistical Analysis

The values of data are expressed as the mean ± SEM (*n* = 6). The statistical analysis of the experiment results was conducted with GraphPad Prism software (GraphPad Software Inc., San Diego, CA, USA). Significance was analyzed using a one-way analysis of variance (ANOVA).

## 5. Conclusions

In this study, histological analysis showed that dieckol ameliorated UVB-induced wrinkle formation and collagen breakdown. Moreover, wrinkle area, depth, and length of the replica skin were improved by dieckol administration. Dieckol inhibited MMP expression via the MAPK signaling pathway and rescued procollagen synthesis by regulating the TGF-β/Smad 2/3 signaling pathway in UVB-irradiated hairless mouse skin. In addition, UVB-induced impairment of hydration factors such as HA, HAS-1/-2, HYAL-1/-2, and filaggrin expression was restored to the control level after dieckol administration. Based on these results, dieckol may be evaluated as a potent nutricosmetic for wrinkled and dehydrated skin.

## Data Availability

Not applicable.

## Supplementary Materials

The following supporting information can be downloaded at: https://www.mdpi.com/article/10.3390/md20120779/s1, Table S1: Primer sequences; Figure S1: Hepatoxicity and renal toxicity of dieckol in hairless mice model; Figure S2: HPLC chromatogram of dieckol at 230 nm and the HPLC analysis conditions of the purity test; Figure S3: 1H NMR spectrum of dieckol; Figure S4: 13C NMR spectrum of dieckol; Figure S5: HPLC–ESI–TOF MS spectrum of dieckol.
