# Supplementary material for: Dieckol Isolated from Eisenia bicyclis Ameliorates Wrinkling and Improves Skin Hydration via MAPK/AP-1 and TGF-β/Smad Signaling Pathways in UVB-Irradiated Hairless Mice
Source: Mar Drugs. 2022 Dec 14;20(12):779. doi: 10.3390/md20120779 (PMC9785544; doi:10.3390/md20120779)
Supplement: Supplementary file 1 [file marinedrugs-20-00779-s001.zip › marinedrugs-2006989-supplementary.pdf]

Supplementary Data

Table S1. Primer sequences

Type	Gene Description	Sequences (5'→3')
Mouse	*COL1A1*	F: CCCAGAACATCACCTATCAC<br>R: GAGGTCTTGGTGGTTTTGTA
	MMP-1	F: AGATCAAGTCCGCTATTTCA<br>R: TTTTTCCAGTCTCTTCCTCA
	MMP-3	F: GTTCTGGGCTATACGAGGGC<br>R: GGCAGCATCGATCTTCTTCA
	MMP-9	F: GGTCTTCCCCAAAGACCTGA<br>R: AGGCCTTTGAAGGTTTGGAA
	HAS1	F: AGTATACCTCGCGCTCCAGA<br>R: AGCAGCAGTAGAGCCCAGAG
	HAS2	F: GACGACGACCTTTACATGAT<br>R: TCTGAAGGCTGTGTACATGA
	HYAL1	F: TACACAGCATGCTCAGAAAG<br>R: AGTGTCTCCATTCCAAACAG
	HYAL2	F: CGAGGACTCACGGGACTGA<br>R: GGCACTCTCACCGATGGTAGA
	Filaggrin	F: AGTGCACTCAGGGGGCTCACA<br>R: CCGGCTTGGCCGTAATGTGT
	GAPDH	F: ATCAAGTGGGGCGATGCTG<br>R: ACCCATGACGAACATGGGG

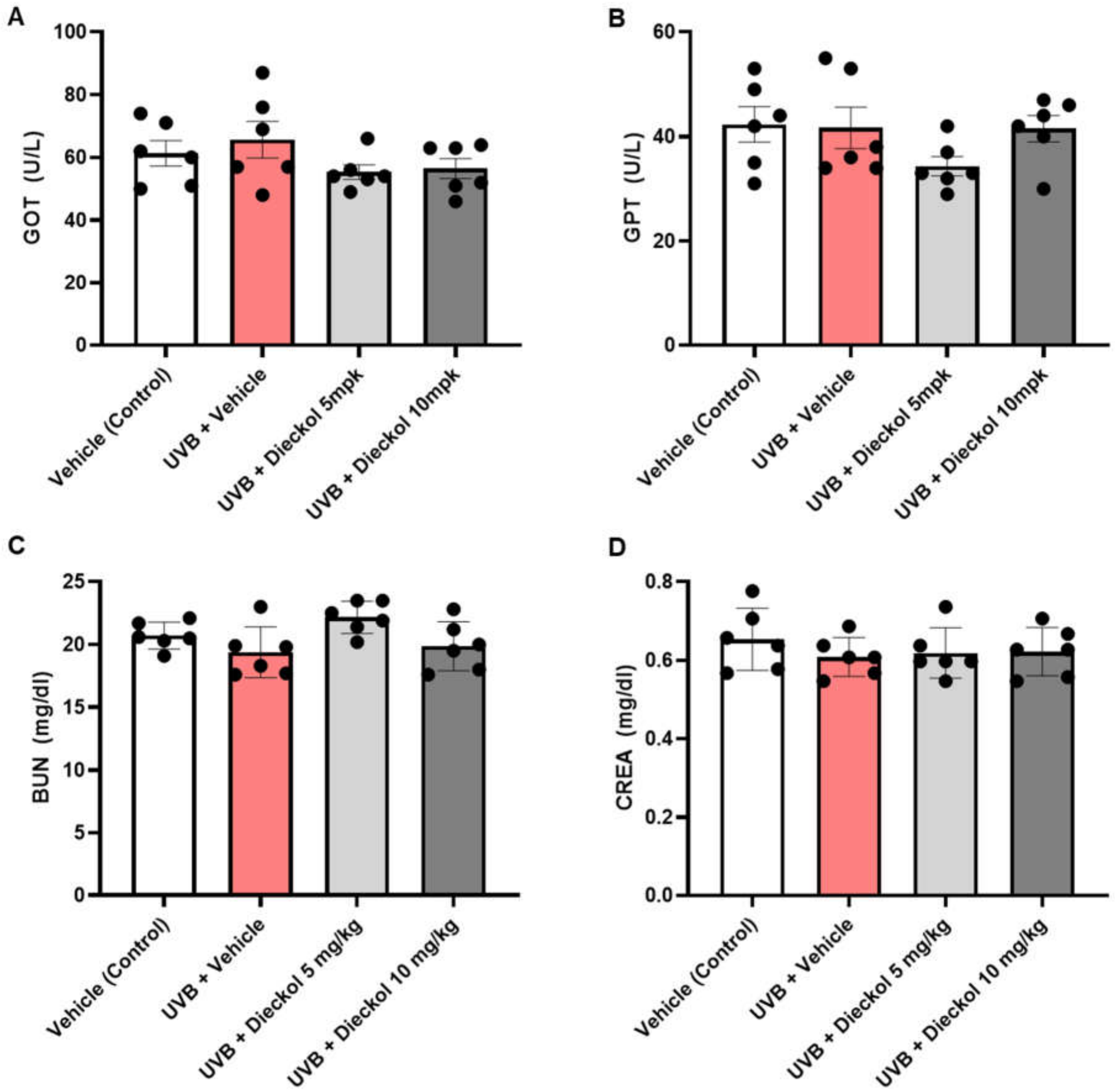

Figure S1. Hepatoxicity and renal toxicity of dieckol in hairless mice model. Biochemistry tests including (**A**) glutamic oxaloacetic transaminase (GOT, AST), (**B**) glutamic pyruvic transaminase (GPT, ALT), (**C**) blood urea nitrogen (BUN), and (**D**) creatinine in plasma of hairless mice model. Data are expressed as the mean ± SEM (n = 6).

A

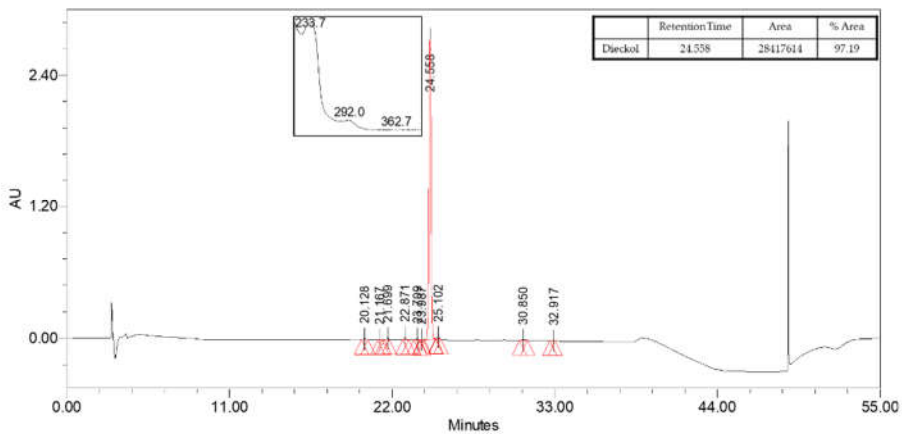

B

Instruments	Waters e2695 Separations Modul e with 2998 PDA detector
Detector	UV 230 nm
Column	Phenomenex Luna C18 column (4.6×250 mm, 5 μm)
Mobile phase	A : acetonitrile<br>B : water (0.1% Formic acid)
Flow rate	1.0 mL/min
Column temp.	30℃
Sample temp.	20℃
Injection volume	10 μℓ

Time	A	B
0	5	95
5	20	80
15	20	80
20	30	70
25	30	70
30	35	65
35	35	65
40	100	0
45	100	0
48	5	95
55	5	95

Figure S2. (**A**) HPLC chromatogram of dieckol at 230 nm and (**B**) the HPLC analysis conditions of purity test.

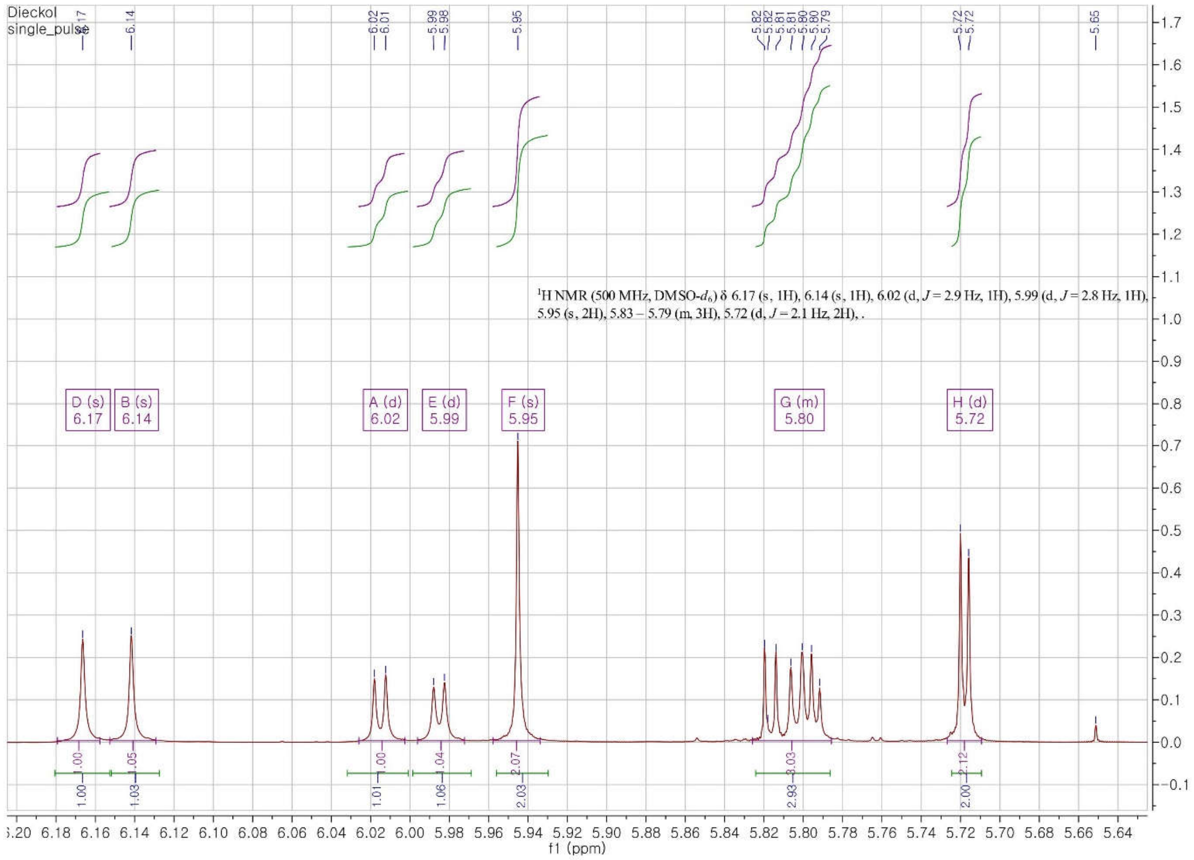

Figure S3. ^{1}H NMR spectrum of dieckol. ^{1}H-NMR data was obtained on a Bruker AM 500 (500 MHz) spectrometer (Bruker, Karlsruhe, Germany) in DMSO-d6 with tetramethylsilane.

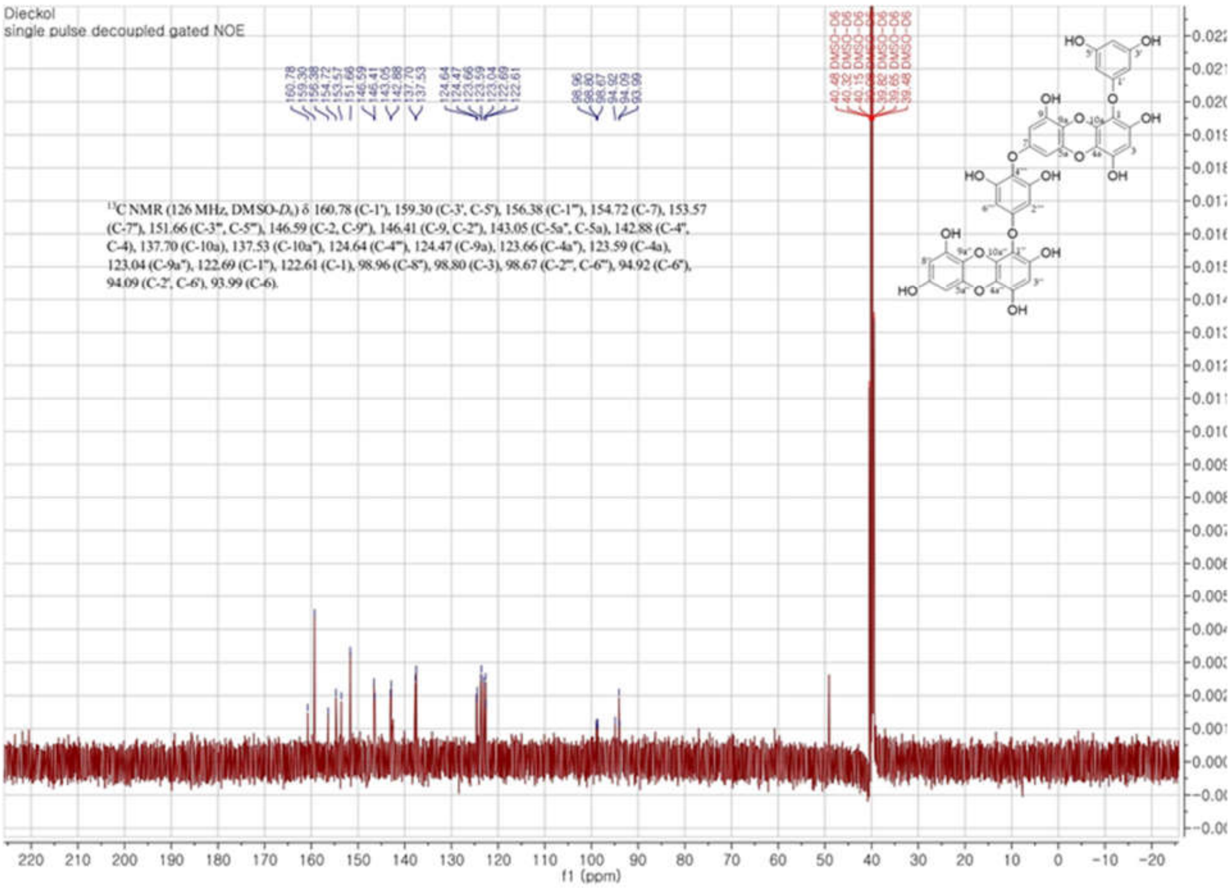

Figure S4. ^{13}C NMR spectrum of dieckol. ^{13}C-NMR data was obtained on a Bruker AM 500 (126 MHz) spectrometer (Bruker, Karlsruhe, Germany) in DMSO-D6 with tetramethylsilane.

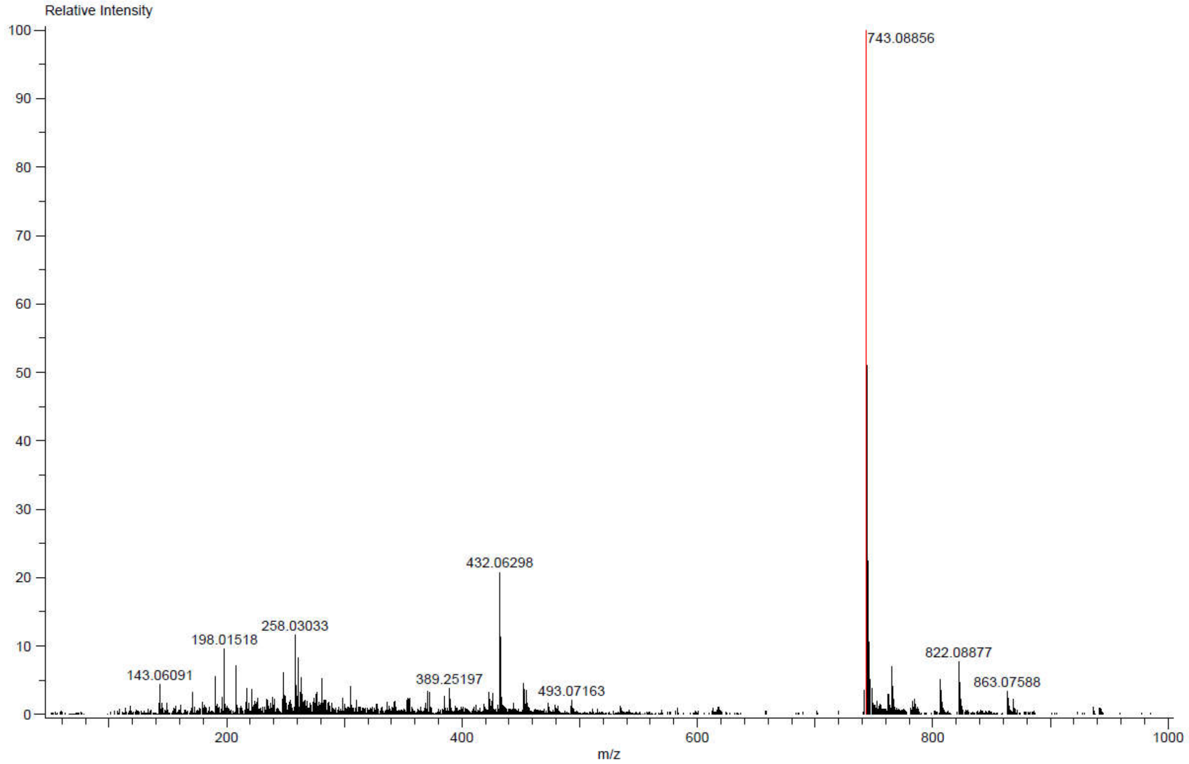

Figure S5. HPLC-ESI-TOF-MS spectrum of dieckol. The operating conditions of the ESI-TOF-MS in the positive ionization mode were: peak voltage is 1000 V; bias voltage is 36 V; ring lens voltage is 15 V; orifice 1 voltage is 80 V; orifice 2 voltage is 7 V; detector voltage is 2000 V; orifice 1 temperature is 80℃; Desolvation temperature is 250℃; N_2 gas flow for nebulizer is 1 L/min; N_2 gas flow for desolvation is 3 L/min; m/z range is 50-1000. Mass scale calibration was accomplished by the standard substance, yokudelna (JEOL).
